# P02-15 Green exercise for well-being: an exploration of psychological responses to physical activity in outdoor and indoor environments

**DOI:** 10.1093/eurpub/ckac095.034

**Published:** 2022-08-29

**Authors:** Laura Scott

**Affiliations:** Fürth, Germany

**Keywords:** green exercise, outdoor, natural environment, psychological outcomes, subjective exercise experience scale

## Abstract

**Background:**

There is an emerging body of knowledge regarding beneﬁts of outdoor activity on well-being, restoration and mood enhancement. This pre-post study explored psychological outcomes in groups carrying out exercise classes in outdoor and indoor settings.

An aim looks to environment in group classes for physical activity programs for population health.

Research Questions: (1) What are the psychological outcomes after a green exercise session (2) Is there a difference in psychological scores between indoor and outdoor exercise programs?

**Methods:**

Two groups of subjects undertaking similar exercises for one hour, in two settings: a ﬁtness studio (N = 19) and a city park (N = 15), subjects completed the Subjective Exercise Experience Scale (SEES) prior to and post classes.

Paired t-tests for dependent groups identiﬁed differences in the three categories of SEES, computed by IMB SPSS Statistics 20.

**Results:**

Exercise in each group improved scores of psychological well-being.

Independent samples t-test showed the outdoor group reported higher psychological well-being markers in the pre-survey. Statistically signiﬁcant difference for “awful” with indoor (M = 2.8421, *SD*=1.64192) and outdoor (M = 1.7333, *SD*=.96115); conditions t(32)=2.32, p=.027. Paired samples t-test showed weak statistical signiﬁcance for improved values for psychological well-being (M = 5.36, *SD*=1.24) and decreased values for psychological distress (M = 1.08, *SD*=1.87) in the indoor group pre to post.

There were no statistically signiﬁcant differences between pre and post survey data, the outdoor environment did not garner higher response scores than the indoor group. Limitations included relatively small sample size, difference in age between outdoor and studio participants, as well as a hot summer in Germany, which may have impacted the perceived well-being scores.

**Conclusions:**

Exercise is beneﬁcial to psychological well-being, regardless of environment. The study did not provide evidence that green exercises elicited greater psychological responses, likely due to study limitations. The outdoor cohort, 50-70 years of age, demonstrated a heightened interest in outdoor programs. Indoor exercise classes should be encouraged as it decreases distress. Green exercise could foster anticipation of a more positive experience yet weather could impact mood outcomes. Intensity, hydration and shade should be prioritized. Future green exercise studies should include objective measures such as age and weather conditions.

